# Follicle-stimulating hormone (FSH) promotes retinol uptake and metabolism in the mouse ovary

**DOI:** 10.1186/s12958-018-0371-9

**Published:** 2018-05-26

**Authors:** Zhuo Liu, Yongfeng Sun, Yanwen Jiang, Yuqiang Qian, Shuxiong Chen, Shan Gao, Lu Chen, Chunjin Li, Xu Zhou

**Affiliations:** 1 College of Animal Science, Jilin University, 5333 Xian Road, Changchun, 130062 Jilin China; 2 College of Animal Science and Technology, Jilin Agricultural University, 2888 Xincheng Street, Changchun, 130118 Jilin China

**Keywords:** Follicle-stimulating hormone, Retinol, Ovary, Granulosa cells

## Abstract

**Background:**

Retinoids (retinol and its derivatives) are required for the development and maintenance of normal physiological functions of the ovary. However, the mechanisms underlying the regulation of ovarian retinoid homeostasis during follicular development remain unclear.

**Methods:**

The present study determined retinoid levels and the expression levels of genes involved in the retinol uptake and its metabolic pathway in the ovaries of follicle-stimulating hormone (FSH)-treated mice and in granulosa cells treated with FSH using ultra performance liquid chromatography (UPLC) combined with quadrupole time-of-flight high-sensitivity mass spectrometry (Q-TOF/HSMS) and real-time PCR analysis.

**Results:**

The levels of total retinoids and retinoic acid (RA) and expressions of retinol-oxidizing enzyme genes alcohol dehydrogenase 1 (*Adh1*) and aldehyde dehydrogenase *(Aldh1a1)* are increased in the ovaries of mice treated with FSH; in contrast, the retinyl ester levels and retinol-esterifying enzyme gene lecithin: retinol acyltransferase (*Lrat*) expression are diminished. In FSH-treated granulosa cells, the levels of retinyl esters, retinaldehyde, and total retinoids are augmented; and this is coupled with an increase in the expressions of stimulated by retinoic acid 6 (*Stra6*) and cellular retinol-binding protein 1 (*Crbp1*), genes in the retinol uptake pathway, and *Adh1*, *Adh7*, and *Aldh1a1* as well as a diminution in *Lrat* expression.

**Conclusions:**

These data suggest that FSH promotes retinol uptake and its conversion to RA through modulating the pathways of retinol uptake and metabolism in the mouse ovary. The present study provides a possible mechanism for the regulation of endogenous RA signaling in the developing follicles.

## Background

Follicles are the functional units of the ovary, with primary functions being oocyte maturation and steroid hormone biosynthesis and secretion. After entering puberty, some follicles begin to grow under the indirect stimulation of gonadotropin-releasing hormone (GnRH) and ultimately culminate in either atresia or ovulation. The processes of follicular development and ovulation are primarily controlled by neuroendocrine activities in the hypothalamus–pituitary–ovary (HPO) axis, although early stages appear to occur independently of the HPO axis. Follicle-stimulating hormone (FSH) and luteinizing hormone (LH), which are released by the pituitary gland, principally control follicular development and ovulation by regulating estradiol (E_2_) secretion and the functions of granulosa and theca cells [1]. In addition to neuroendocrine mechanisms, cell-cell communications between oocyte and somatic (granulosa and theca) cells play critical roles in the initiation and coordination of somatic cell and oocyte differentiation [2–5]. Paracrine interactions between oocyte and their surrounding granulosa cells during oocyte and follicular development ensure proper coordination of oocyte and somatic cell functions [2, 4, 5]. It has been well established that the retinoid pathway plays a fundamental role in maintaining the normal ovarian function [6]. Kawai et al. [7] reported that retinoic acid (RA) in antral follicles was required for FSH-regulated granulosa cell differentiation and ovarian reproductive competence, and that retinoid deficiency prevented the development of oocytes and reduced the number of ovulated oocytes in mice. RA is also required for both nuclear and cytoplasmic maturation of mouse and bovine oocytes [8, 9], and can stimulate steroidogenesis, such as for testosterone synthesis in human theca cells and estradiol synthesis in mouse granulosa cells [6, 10]. In addition, ovarian retinoid levels vary with the estrous cycle [11], and the concentration of retinol is greater in the fluid of dominant follicles relative to that of small follicles [12, 13]. However, the regulatory mechanisms underlying ovarian retinoid homeostasis are not currently fully understood.

Retinol (vitamin A) and its derivatives (retinyl esters, retinal, and RA) are collectively known as retinoids. It is generally understood that most retinoids are taken up by extrahepatic tissues from retinol-binding protein 4 (RBP4)-bound retinol in the circulation through transmembrane-spanning protein stimulated by RA 6 (STRA6), which acts as the cell surface receptor for RBP4 and can facilitate the transport of retinol from RBP4-retinol complexes into cells [14]. To be biologically active, intracellular retinol must first be oxidized to retinaldehyde and then to RA and a large number of enzymes and binding proteins are involved in these processes. Upon entering cells, retinol is bound by free cellular retinol-binding protein 1 (CRBP1). STRA6, as a bidirectional transporter of retinol, is potentially involved in maintaining intracellular retinoid homeostasis along with RBP4 and CRBP1 [14–17]. Within cells, retinol can either be converted to retinaldehyde and RA via 2 enzymatic steps or be stored in cells as retinyl esters catalyzed by lecithin: retinol acyltransferase (LRAT). First, retinol can be oxidized to retinaldehyde by alcohol dehydrogenases (ADHs, such as ADH1 and ADH7), and then retinal can be oxidized to RA by aldehyde dehydrogenases (ALDHs, such as ALDH1A1) [18]. Most of the cellular actions of retinoids are thus realized due to the transcriptional regulatory activity of RA, which binds nuclear RA receptors (RARs: RARα, RARβ, and RARγ) as well as the peroxisome proliferator activated receptor β/δ (PPARβ/δ); RARs and PPARβ/δ then associate with retinoid X receptors (RXRs: RXRα, RXRβ, and RXRγ) to form heterodimers and combine with RA response elements (RAREs) or peroxisome proliferator response elements (PPREs) within the promoters of retinoid-responsive genes [6, 19]. It was reported that the partition of RA between the two signaling pathways exerts opposing action on cell growth and apoptosis and that the alternative pathways are coordinated by cellular RA-binding protein 2 (CRABP2) and fatty acid–binding protein 5 (FABP5), which bind and transport RA to RARs and PPARβ/δ, respectively [19, 20]. In addition to the classical direct nuclear receptor signaling pathways, RA stimulates rapid, nongenomic signaling events by inducing kinase phosphorylation and activation via binding to extra-nuclear RARs, which subsequently leads to downstream nuclear effects on transcription [21].

The present study was aimed to investigate the regulatory mechanisms underlying ovarian retinoid accumulation and metabolism. To this end, we examined retinoid levels using ultra performance liquid chromatography (UPLC) combined with quadrupole time-of-flight high-sensitivity mass spectrometry (Q-TOF/HSMS) in ovaries of FSH-treated mice and in follicular granulosa cells treated with FSH. We also determined the expressions of genes of enzymes and binding proteins involved in retinol uptake and metabolism. Our data showed that FSH promoted retinol uptake and its conversion to RA in both mouse ovaries in vivo and in granulosa cells cultured in vitro.

## Material and methods

### Animals

Three-week-old immature female BALB/c mice were obtained from the Medical Department of Jilin University (Changchun, China), raised in an environment with controlled temperature (22–24 °C) and humidity (60–70%) in a 12-h light/dark cycle, and provided with food and water ad libitum. They were injected intraperitoneally with a single dose of FSH (10 IU/mouse; Ningbo Second Hormone Factory, Ningbo, China) [22–24] and sacrificed via cervical dislocation 24 or 48 h after injection. Ovary tissues were rapidly collected and stored at − 80 °C. All animal studies were conducted in strict accordance with the protocol approved by the Animal Care and Use Committee of Jilin University.

### Isolation of follicular granulosa cells and culture in vitro

Primary granulosa cells were isolated from immature female mouse ovaries, as described previously [25, 26]. In brief, mice were sacrificed via cervical dislocation after being anesthetized, and the follicles were isolated with no. 5 fine needles. The follicles were then treated with trypsin (Hyclone, USA) for 1 h and filtered using a 100-μm filter (Life Technologies, USA). The isolated granulosa cells were cultured in Dulbecco’s Modified Eagle Medium/F12 1:1 (Hyclone, USA) supplemented with 10% fetal bovine serum (Hyclone, USA), 1% insulin–transferrin–selenium (Sigma, USA), and 1% antibiotics (Hyclone, USA) at 37 °C in an atmosphere of 5% CO_2_ in compressed air at high humidity. Twenty-four hours later, non-adherent cells were removed and adherent cells were treated with FSH (100 IU/L) in the presence of all-*trans*-retinol (1 μM, approximately to the concentration of retinol in 100% serum; Sigma, USA).

### Sample preparation and liquid chromatography–mass spectrometry (LC-MS) analysis

Ovary tissues (50 mg) and granulosa cells (5 × 10^6^) were each homogenized in 800 μL of methanol with an internal standard (5 μg/ml, DL-o-chlorophenylalanine), the homogenates were centrifuged at 13,000 rpm for 15 min, and the supernatants (200 μl) were collected. LC-MS was carried out on a Waters Acquity™ UPLC system (Waters, USA) coupled with a Waters XevoTM G2 QTOF-MS (Waters, UK). Chromatography was performed on an Acquity UPLC high strength silica (HSS) T3 column (2. 1 mm × 100 mm, 1. 8 μm, UK) at 40 °C. The mobile phases were water (A) and acetonitrile (B) containing 0.1% formic acid. The optimized elution conditions for LC are shown in Table 1. 6-μL sample solution was injected for each run, and MS analysis was performed on a mass spectrometer XevoTM G2 QTof (Waters, UK). For the positive electrospray mode, the capillary and cone voltage were set at 1.4 kV and 40 V, respectively. The desolvation gas flow was set to 600 L/h at 350 °C, the cone gas flow was set to 50 L/h and the source temperature was set to 120 °C. The collision and ion energies were 10–40 V and 1 V, respectively. The data acquisition rate was set to 0.1 s, with a 0.1 s interscan delay; the scan range was from 50 to 1500 m/z. Rutin solution was used as the lockmass to ensure accuracy and reproducibility. All the acquisition and analysis of data were performed using Waters MassLynx v4.1 software.

**Table 1:** UPLC elution conditions

Time (min)	Flow rate (ml/min)	Pressure limit (bar)	Solv Ratio B (%)
0	0.35	800	5
1	0.35	800	5
6	0.35	800	20
9	0.35	800	50
13	0.35	800	95
15	0.35	800	95

### Total RNA extraction and real-time quantitative PCR assay

Total RNA from ovarian tissues and granulosa cells cultured in vitro was extracted using an RNAprep pure Micro Kit (Tiangen, Beijing, China) and reverse transcribed into cDNA using a PrimeScript RT reagent kit (Takara, Japan) according to the manufacturer’s instructions. Real-time PCR was performed on a sequence-detection system (Agilent Technologies, USA) using the SYBR Premix Ex TaqII kit (Takara, Japan), with *β-Actin* used as an internal reference. The relative mRNA expression levels were calculated using the 2^−ΔΔCt^ method. All primers were obtained from Sangon Biotech (Shanghai, China), and information for the primers is shown in Table 2. All experiments were repeated at least three times.

**Table 2:** Primer list

Gene	Forward	Reverse	Size (bp)	Annealing temperature
*β-Actin*	5′-TCTGGCACCACACCTTCTA-3′	5′-AGGCATACAGGGACAGCAC-3′	180	60
*Stra6*	5′-AGGGCCCTGGAAGCTACTG-3′	5’-AGGCCAGCAAGGAGTAGTC-3′	197	60
*Crbp1*	5′-GCCTTACGCAAAATCGCCAA-3′	5′-ACAGTGGTCATGCACTTGCG-3′	176	60
*Adh1*	5′-TTGGCTGTAAAGCAGCAGGA-3′	5′-CATGGGGTTCATGGAGAGGT-3′	293	60
*Adh7*	5′-CTGGTGCCTCCAGGATCATT-3′	5′-CCCAGTGAAGAGCAGCATTG-3′	293	60
*Aldh1a1*	5′-CCCGGATTTTTGTTGAGGAG-3′	5′-GAGAACACTGTGGGCTGCAC-3′	244	60
*Lrat*	5′-AGGTGACACGGACCCATTTT-3′	5′-CTGCTCCGTAGGCAAAGTCC-3′	205	60

### Statistical analyses

Statistical analyses of the data were conducted via independent sample t-tests or one-way ANOVA (Fig. 2a), followed by Tukey’s test. Differences were considered to be significant at *P* < 0.05. All the statistical analyses were performed using SPSS 22.0 for Windows (StatSoft, USA).

## Results

### Retinoid levels in ovaries of mice treated with FSH

**Fig. 1:**
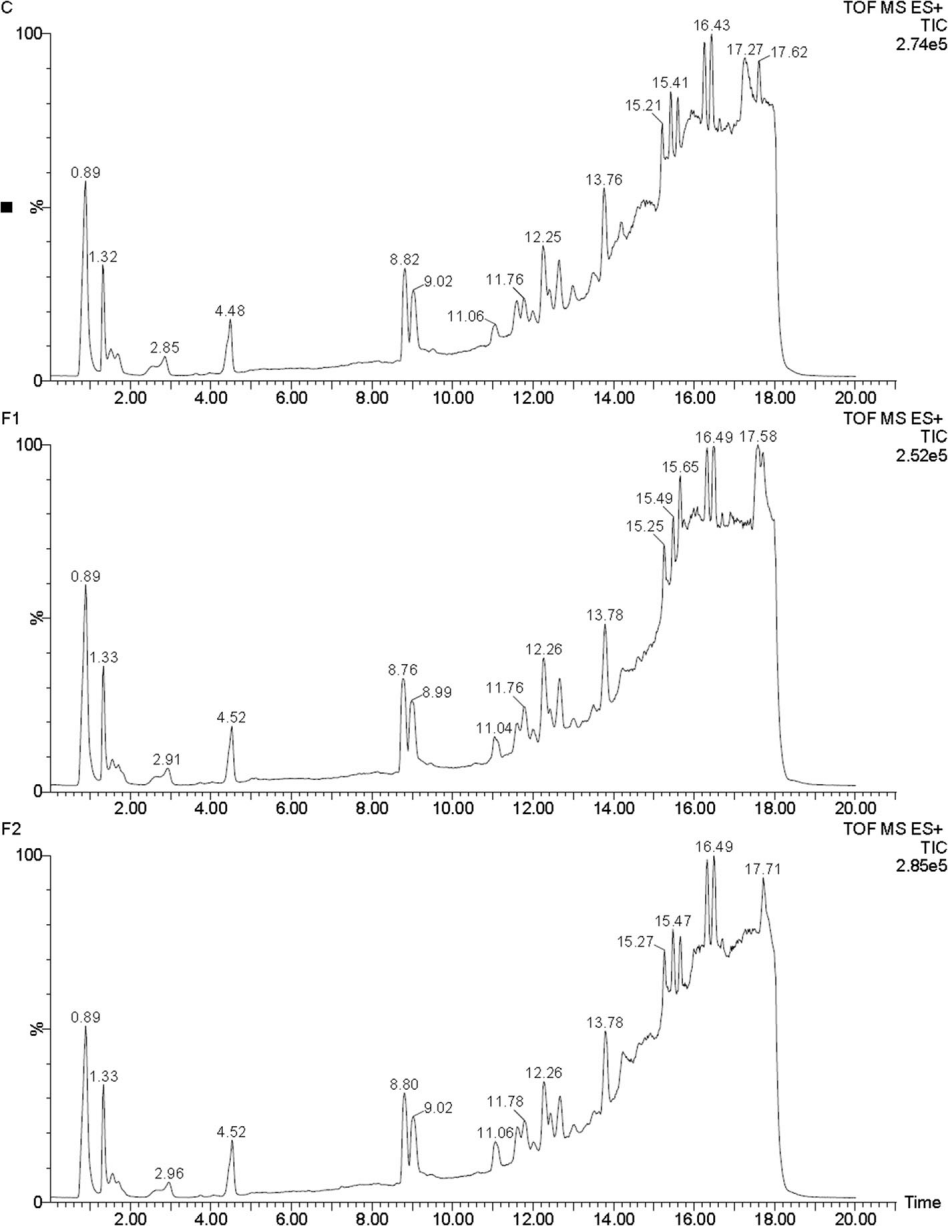
Representative LC-MS TICs of samples from the ovaries of mice treated with (C) or without FSH for 24 (F1) or 48 h (F2)

**Fig. 2:**
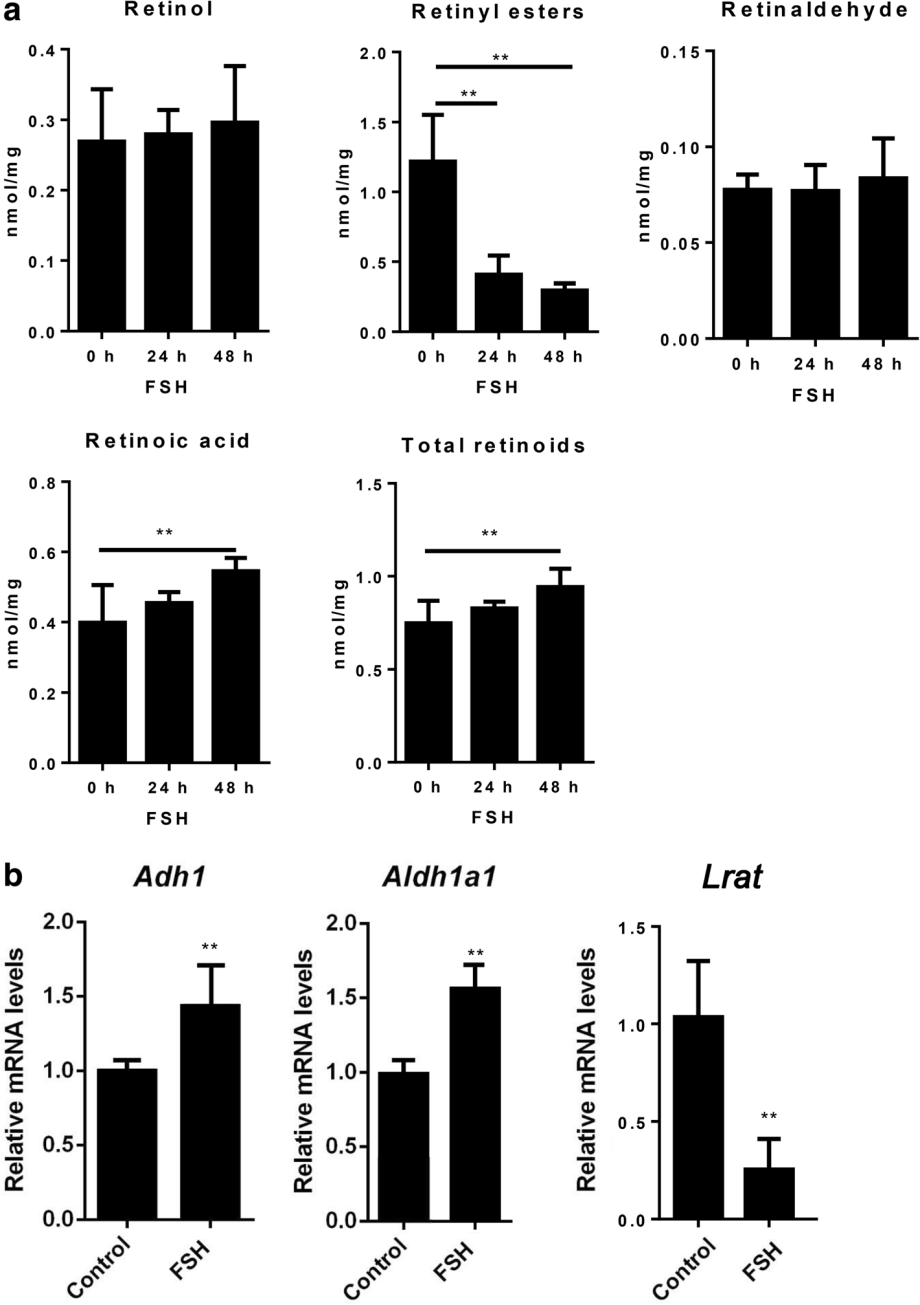
Effects of FSH on retinol uptake and metabolism in the mouse ovaries. **a** Semi-quantitative LC-MS analysis of retinoid levels in ovaries from untreated mice or mice treated with FSH for 24 or 48 h. **b** Real-time PCR analyses of the expression of genes in retinol uptake and metabolism pathways in ovaries from untreated mice or mice treated with FSH for 48 h. Data are presented as means ± SEM, *n* = 5. *p < 0.05, **p < 0.01

The retinoid levels in ovaries of mice left untreated or treated with FSH were examined using semi-quantitative LC-MS analysis. Representative LC-MS total-ion chromatograms (TICs) of the samples are displayed in Fig. 1. The retinoid metabolites were identified by searching against the METLIN Metabolite Database (http://metlin.scripps.edu/) and Human Metabolome Database (HMDB, http://www.hmdb.ca/) and comparing the accurate masses or mass-to-charge ratios (m/z). The retinoid levels were calculated using the formula C_x_ = S_x_/S_s_*ρs*V/m/M_x_ (μmol/mg) for tissue samples or = S_x_/S_s_*ρ_s_/M_x_ (umol/ml) for cell samples, where S_x_ and S_s_ indicate integral areas of the peaks of metabolite X and internal standard, respectively; ρ_s_ indicates the concentrations of internal standard (ug/ml); V indicates the volume of extraction solvent (ml); M_x_ indicats the molar mass of metabolite X; and m indicates the weight of tissue samples (mg). The results showed that the levels of RA and total retinoids increased in the ovaries of mice treated with FSH for 48 h; in contrast, retinyl esters diminished (Fig. 2a).

### Expression of genes in retinol uptake and metabolism pathways in ovaries of mice treated with FSH

We further determined the expression of the genes involved in retinol uptake and metabolism. The data showed that the expression of the alcohol dehydrogenase gene *Adh1* and aldehyde dehydrogenase gene *Aldh1a1* increased with FSH, but that the expression of the lecithin: retinol acyltransferase gene *Lrat* decreased (Fig. 2b).

### Retinoid levels in follicular granulosa cells treated with FSH

**Fig. 3:**
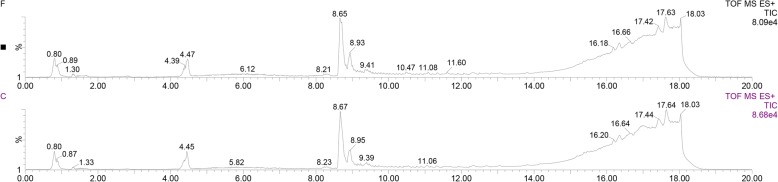
Representative LC-MS TICs of samples from granulosa cells treated with (F) or without (C) FSH for 24 h

**Fig. 4:**
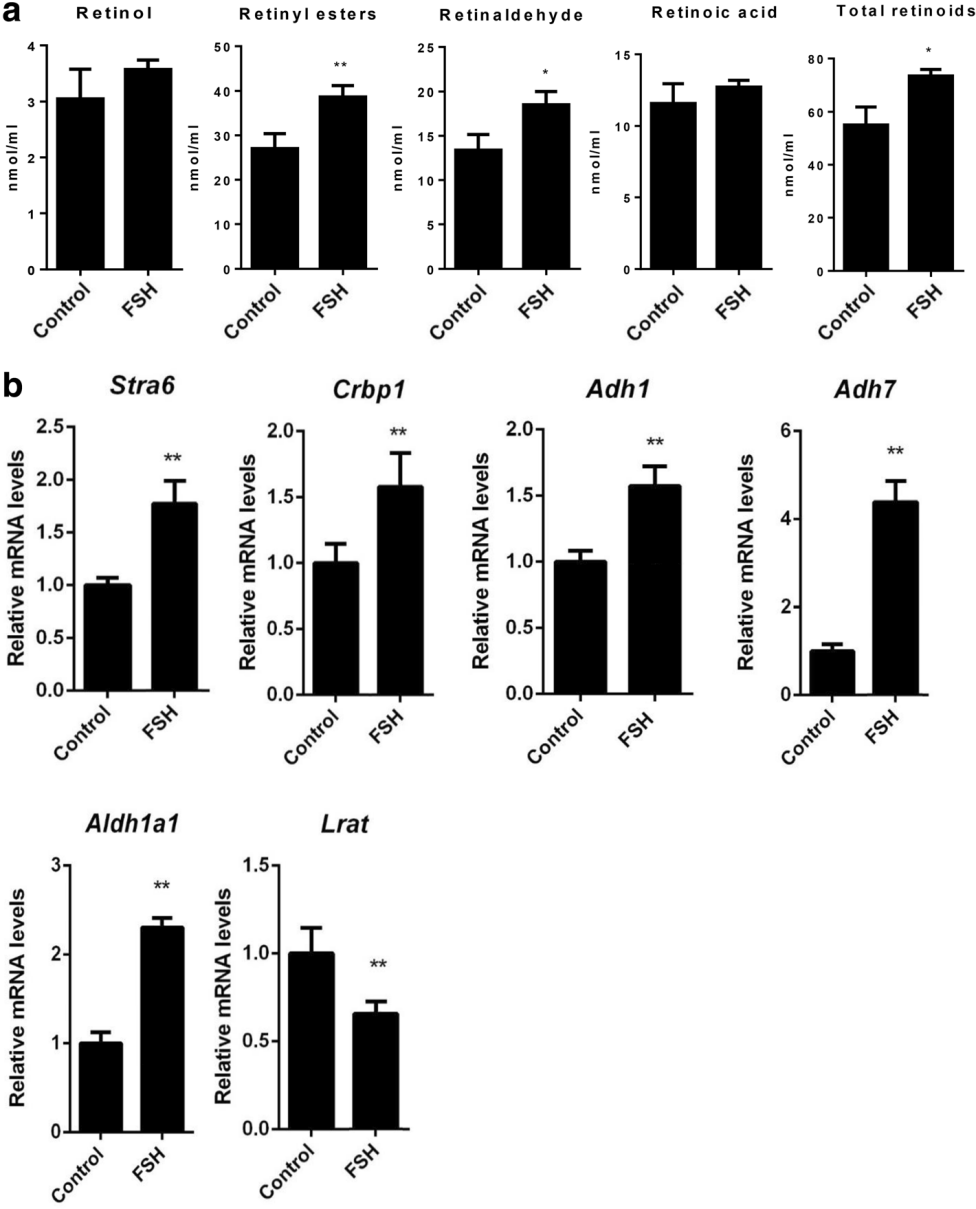
Effects of FSH on retinol uptake and metabolism in granulosa cells. **a** Semi-quantitative LC-MS analysis of retinoid levels in mouse follicular granulosa cells in the presence or absence of FSH for 24 h. **b** Real-time PCR analyses of the expression of genes in retinol uptake and metabolism pathways in mouse follicular granulosa cells in the presence or absence of FSH for 24 h. Data are presented as means ± SEM, *n* = 4. *p < 0.05, **p < 0.01

Since granulosa cells are the main cell types regulated by FSH in the ovary, we further evaluated the effects of FSH on retinoid levels in granulosa cells. The TICs of the samples are displayed in Fig. 3. The results showed that the levels of retinyl esters, retinaldehyde, and total retinoids increased significantly in cells treated with FSH for 24 h compared with untreated controls (Fig. 4a). The levels of retinol and RA also tended to show an increase (though not significant) (Fig. 4a).

### Expression of genes in retinol uptake and metabolism pathways in ovarian follicular cells treated with FSH

We also determined the expression of genes involved in retinol uptake and metabolism in granulosa cells. The results showed that the mRNA levels for *Stra6*, *Crbp1*, *Adh1*, *Adh7*, and *Aldh1a1* were increased when treated with FSH; however, that of *Lrat* decreased (Fig. 4b).

## Discussion

Results from previous studies have shown that de novo synthesized RA, a potent, bioactive member of the retinoid signaling family, plays crucial roles in ovarian functions [7–10, 27]. RA can enhance LHR expression, stimulate steroidogenesis in granulosa cells and promote oocyte mutation. In addition, the levels of retinoids vary during the estrous cycle and follicular growth, with higher levels in the follicular fluids of large antral follicles [11–13]. However, the mechanisms underlying the regulation of ovarian retinoid accumulation and RA biosynthesis during follicular development remain unclear.

FSH, the primary reproductive hormones that control follicular development and ovarian functions, may be involved in the regulation of ovarian retinoid accumulation and metabolism. Our previous data showed that FSH can stimulate RBP4 expression in developing follicles [23]. RBP4, which acts as the mediator for the systemic and intercellular transport of retinol, plays an important role in the cellular retinol influx, efflux, and exchange of retinol [28]; and seems to play a role in intercellular transport and accumulation of retinol in follicular fluids of dominant follicles [12, 23]. Besides, other studies have also shown that FSH stimulated the expression of several enzymes and binding proteins that are involved in RA synthesis in granulosa and Sertoli cells [7, 23, 29].

In the present study, using semi-quantitative LC-MS analyses, we demonstrated that FSH increased the levels of total retinoids and RA in mouse ovaries in vivo*.* The results from real-time PCR analyses showed correspondingly that FSH stimulated the expression of ADH1 and ALDH1 genes, which catalyze the conversion of retinol to retinal and retinal to RA, respectively [6]. In contrast, FSH decreased the levels of retinyl esters and inhibited the expression of LRAT, which catalyzes the esterification of retinol [6]. Retinyl esters, such as retinyl palmitate, are thought to be a storage form of retinol [15]; under certain conditions, these retinyl esters are hydrolyzed by cells to produce retinol and bioactive retinoids such as RA. Thus, FSH likely promotes retinol uptake and the conversion of retinol to retinal and RA, and inhibits the esterification of retinol in the mouse ovary in vivo.

As follicular granulosa cells constitute the primary FSH-responsive cell types in the ovary, we employed mouse granulosa cells in primary culture as an in vitro cell model to further confirm the effect of FSH on retinol uptake and metabolism in the mouse ovary. The results showed that FSH increased the levels of total retinoids and retinal; and that FSH also stimulated the gene expression of STRA6 and CRBP1 (which are thought to play important roles in retinol uptake by cells [14–17]), ADH1 and ADH7 (which catalyze the conversion of retinol to retinal [6]), and ALDH1A1 (which catalyzes the conversion of retinal to RA [6]). Therefore, FSH also enhances the uptake and metabolism of retinol in granulosa cells, though the increase in RA levels was not significant. The increase in retinyl ester levels may be caused by the quick uptake of retinol into cells under the stimulation of FSH, which may then result in the accumulation of retinyl esters. Another study showed that FSH can also increase retinyl ester levels in the presence of physiological concentration of retinol (i.e. 1 μM) in Sertoli cells cultured in vitro [29].

Kawai et al. [7] reported that STRA6, the transmembrane-spanning bidirectional transporter of retinol, was primarily distributed in granulosa cells of large antral follicles. Besides, CRBP1, which also plays an important role in the transportation of retinol into cells, was also reported to be expressed in granulosa cells [12]. ADH1 and ALDH1A1 were also expressed in granulosa cells, though higher expression levels were observed in the theca cell layer [7]. Thus, it is likely that granulosa cells could take up retinol and transform it into retinal and RA. RA synthesized from retinol exerts fundamental actions in granulosa cell differentiation and functions [6]; meanwhile, it may then be secreted from the granulosa cells and delivered to the developing oocytes as a paracrine factor to assist oocyte development and maturation through its receptor RARs. It has been proved that RARs are expressed in all cell types in developing follicles and that RA can promote the nuclear and cytoplasmic maturation of oocytes cultured in vitro [6].

In this study, the increase in RA levels in FSH-stimulated granulosa cells cultured in vitro was not significant but the RA levels increased significantly in the ovaries of FSH-injected mice. This inconsistency may be caused by two possibilities. First, the cells were treated for 24 h in the presence of physiological concentrations of retinol; the time might be too long and the increased RA might be degraded. In another similar study, in which Sertoli cells (one major FSH-responsive cell type in testis) were pre-cultured with FSH for 24 h, followed by incubation with retinol, Guo et al. [29] showed that total RA increased significantly within 2 h and by 12 h no difference was seen from the control. Besides, the cell culture condition may be not very mimic to in vivo environment; in the physical condition, there are many other factors (for example testosterone) existing in the surroundings of granulosa cells.

## Conclusion

In conclusion, in the present study, we demonstrated that FSH promoted retinol uptake and its conversion to RA in the mouse ovary. This information is significant when elucidating the mechanisms by which production of endogenous RA-signaling molecules are regulated in the developing follicles.

## Abbreviations

ADH: Alcohol dehydrogenase
ALDH: Aldehyde dehydrogenases
CRBP: Cellular retinol-binding protein
FSH: Follicle-stimulating hormone
HPLC: Ultra performance liquid chromatography
LRAT: Lecithin:retinol acyltransferase
MS: Mass spectrometry
RA: Retinoic acid
RAR: Receptor
RBP4: Retinol-binding protein 4
RXR: Retinoid X receptor
STRA6: Stimulated by RA 6

## References

[1] Walker DM, Gore AC (2011). Transgenerational neuroendocrine disruption of reproduction. Nat Rev Endocrinol.

[2] Thomas FH, Vanderhyden BC (2006). Oocyte-granulosa cell interactions during mouse follicular development: regulation of kit ligand expression and its role in oocyte growth. Reprod Biol Endocrinol.

[3] Eppig JJ (2018). Reproduction: oocytes call, Granulosa Cells Connect. Curr Biol.

[4] Canipari R (2000). Oocyte--granulosa cell interactions. Hum Reprod Update.

[5] Orisaka M, Tajima K, Tsang BK, Kotsuji F (2009). Oocyte-granulosa-theca cell interactions during preantral follicular development. J Ovarian Res.

[6] Jiang YW, Li CJ, Chen L, Wang FG, Zhou X (2017). Potential role of retinoids in ovarian physiology and pathogenesis of polycystic ovary syndrome. Clin Chim Acta.

[7] Kawai T, Yanaka N, Richards JS, Shimada M (2016). De novo-synthesized retinoic acid in ovarian antral follicles enhances FSH-mediated ovarian follicular cell differentiation and female fertility. Endocrinology.

[8] Ikeda S, Kitagawa M, Imai H, Yamada M (2005). The roles of vitamin A for cytoplasmic maturation of bovine oocytes. J Reprod Dev.

[9] Tahaei LS, Eimani H, Yazdi PE, Ebrahimi B, Fathi R (2011). Effects of retinoic acid on maturation of immature mouse oocytes in the presence and absence of a granulosa cell co-culture system. J Assist Reprod Genet.

[10] Wickenheisser JK, Nelson-DeGrave VL, Hendricks KL, Legro RS, Strauss JF, McAllister JM (2005). Retinoids and retinol differentially regulate steroid biosynthesis in ovarian theca cells isolated from normal cycling women and women with polycystic ovary syndrome. J Clin Endocrinol Metab.

[11] Haliloglu S, Baspinar N, Serpek B, Erdem H, Bulut Z (2002). Vitamin A and beta-carotene levels in plasma, corpus luteum and follicular fluid of cyclic and pregnant cattle. Reprod Domest Anim.

[12] Brown JA, Eberhardt DM, Schrick FN, Roberts MP, Godkin JD (2003). Expression of retinol-binding protein and cellular retinol-binding protein in the bovine ovary. Mol Reprod Dev.

[13] Schweigert FJ, Zucker H (1988). Concentrations of vitamin A, beta-carotene and vitamin E in individual bovine follicles of different quality. J Reprod Fertil.

[14] Kawaguchi R, Yu J, Honda J, Hu J, Whitelegge J, Ping P (2007). A membrane receptor for retinol binding protein mediates cellular uptake of vitamin A. Science.

[15] D'Ambrosio DN, Clugston RD, Blaner WS (2011). Vitamin A metabolism: an update. Nutrients.

[16] Isken A, Golczak M, Oberhauser V, Hunzelmann S, Driever W, Imanishi Y (2008). RBP4 disrupts vitamin A uptake homeostasis in a STRA6-deficient animal model for Matthew-wood syndrome. Cell Metab.

[17] Kim YK, Wassef L, Hamberger L, Piantedosi R, Palczewski K, Blaner WS (2008). Retinyl ester formation by lecithin: retinol acyltransferase is a key regulator of retinoid homeostasis in mouse embryogenesis. J Biol Chem.

[18] Conaway HH, Henning P, Lerner UH (2013). Vitamin A metabolism, action, and role in skeletal homeostasis. Endocr Rev.

[19] Jiang Y, Chen L, Taylor RN, Li C, Zhou X (2018). Physiological and pathological implications of retinoid action in the endometrium. J Endocrinol.

[20] Delva L, Bastie JN, Rochette-Egly C, Kraiba R, Balitrand N, Despouy G (1999). Physical and functional interactions between cellular retinoic acid binding protein II and the retinoic acid-dependent nuclear complex. Mol Cell Biol.

[21] Rochette-Egly C (2015). Retinoic acid signaling and mouse embryonic stem cell differentiation: cross talk between genomic and non-genomic effects of RA. Biochimica Et Biophysica Acta-Molecular And Cell Biology Of Lipids.

[22] Zhou J, Li C, Yao W, AA M, Huo L, Liu H, et al. Hypoxia-inducible factor-1alpha-dependent autophagy plays a role in glycolysis switch in mouse granulosa cells. Biol Reprod. 2018; 10.1093/biolre/ioy061. 10.1093/biolre/ioy061 29546328

[23] Jiang Y, Zhao Y, Chen S, Chen L, Li C, Zhou X (2018). Regulation by FSH of the dynamic expression of retinol-binding protein 4 in the mouse ovary. Reprod Biol Endocrinol.

[24] Gong S, Sun GY, Zhang M, Yuan HJ, Zhu S, Jiao GZ (2017). Mechanisms for the species difference between mouse and pig oocytes in their sensitivity to glucorticoids. Biol Reprod.

[25] Liang N, Xu YL, Yin YM, Yao GD, Tian H, Wang GS (2011). Steroidogenic Factor-1 is required for TGF-beta 3-mediated 17 beta-estradiol synthesis in mouse ovarian granulosa cells. Endocrinology.

[26] Yao GD, Yin MM, Lian J, Tian H, Liu L, Li X (2010). MicroRNA-224 is involved in transforming growth factor-beta-mediated mouse granulosa cell proliferation and granulosa cell function by targeting Smad4. Mol Endocrinol.

[27] Kipp JL, Golebiowski A, Rodriguez G, Demczuk M, Kilen SM, Mayo KE (2011). Gene expression profiling reveals Cyp26b1 to be an activin regulated gene involved in ovarian granulosa cell proliferation. Endocrinology.

[28] Kawaguchi R, Zhong M, Kassai M, Ter-Stepanian M, Sun H (2015). Vitamin a transport mechanism of the multitransmembrane cell-surface receptor STRA6. Membranes (Basel).

[29] Guo X, Morris P, Gudas L (2001). Follicle-stimulating hormone and leukemia inhibitory factor regulate Sertoli cell retinol metabolism. Endocrinology.

